# An Investigation of the Effects of Melatonin and Vitamin D on the Ovaries of a Rat Model of Premature Ovarian Failure Induced by Cyclophosphamide

**DOI:** 10.3390/ijms26167772

**Published:** 2025-08-12

**Authors:** Özdem Karaoğlan, Yurdun Kuyucu, Dilek Şaker, Gülçin Dağlıoğlu, Özgül Tap

**Affiliations:** Department of Histology and Embryology, Faculty of Medicine, Cukurova University, Adana 01170, Turkey; ykuyucu@cu.edu.tr (Y.K.); dsaker@cu.edu.tr (D.Ş.); gdaglioglu@cu.edu.tr (G.D.); otap@cu.edu.tr (Ö.T.)

**Keywords:** folliculogenesis, premature ovarian failure, cyclophosphamide, PTEN, FOXO3a, AMH

## Abstract

In this study, we evaluated the protective effects of combined melatonin and vitamin D3 treatment on ovarian reserve and tissue architecture in a cyclophosphamide-induced premature ovarian failure (POF) rat model. Forty-nine adult female rats were randomly assigned to seven groups, including intact control (group 1), single-agent control (groups 2 and 3), POF (group 4), and POF + treatment (groups 5, 6, and 7) groups. Cyclophosphamide exposure led to elevated FSH and LH levels, reduced estradiol and progesterone levels, extensive follicular atresia, stromal fibrosis, and the marked degeneration of the ovarian ultrastructure. Additionally, the expression levels of PTEN, FOXO3a, and AMH were significantly downregulated, while caspase-3 and TNF-α immunoreactivities were increased. Notably, co-treatment with melatonin and vitamin D3 preserved primordial and growing follicle populations, restored hormonal balance, reduced stromal fibrosis, and attenuated apoptosis and inflammation markers. These findings highlight the potential of combined melatonin and vitamin D3 therapy as a fertility-preserving strategy that functions by mitigating chemotherapy-induced ovarian injury through multi-pathway modulation.

## 1. Introduction

Premature ovarian failure (POF) is a condition characterized by amenorrhea and hypergonadotropic hypogonadism resulting from the loss of ovarian function before the age of 40. It is typically defined by elevated levels of FSH and LH, along with decreased estradiol (E2) concentrations, and constitutes a significant cause of infertility [1] POF may occur spontaneously; however, iatrogenic factors such as pelvic radiotherapy and chemotherapy are among the most common etiological causes [2]. Cyclophosphamide, an alkylating agent widely used as a chemotherapeutic and immunosuppressive drug, impairs ovarian function by depleting the follicular pool, ultimately leading to early menopause and infertility [3].

Although chemotherapy-induced ovarian failure has been demonstrated in various studies, the underlying mechanisms remain incompletely understood. Some studies suggest that the loss of dormant primordial follicles through apoptosis initiates this process, whereas others propose that excessive follicular activation and the subsequent depletion of the follicle pool are the primary contributors [4,5]. During folliculogenesis, stimulatory and inhibitory genes operate in a finely regulated balance. PTEN and FOXO3a are key negative regulators of the PI3K/AKT signaling pathway and play crucial roles in keeping primordial follicles in a quiescent state. Anti-Müllerian hormone (AMH), secreted by granulosa cells of growing follicles, inhibits the further activation of new follicles and is clinically used as a biomarker of ovarian reserve [6].

Melatonin, a hormone synthesized by the pineal gland, plays a role in the regulation of reproductive function and gonadal steroidogenesis. It protects cellular integrity by scavenging free radicals and counteracting oxidative damage induced by reactive oxygen species [7]. Similarly, vitamin D exerts beneficial effects on follicular development, steroidogenic enzyme activity, modulation of immune responses, and implantation success. Vitamin D enhances AMH production, increases FSH sensitivity, and regulates the expression of steroidogenic enzymes such as aromatase and 17β-hydroxysteroid dehydrogenase (17β-HSD) [8]. Low levels of vitamin D have been associated with infertility, polycystic ovary syndrome (PCOS), premature ovarian failure (POF), and ovarian cancer [9]. Moreover, vitamin D has been shown to influence ovarian reserve markers by modulating AMH signaling pathways in granulosa cells [10]. The present study aimed to investigate the effects of the combined administration of melatonin and vitamin D as a pharmacological strategy for preserving fertility in a rat model of cyclophosphamide-induced ovarian damage. In particular, this study focused on follicular histology and the inhibitory factors PTEN, FOXO3a, and AMH, which play a critical role in preserving the ovarian follicular reserve by suppressing premature follicular development.

## 2. Results

### 2.1. Biochemical Findings

**Figure 1:**
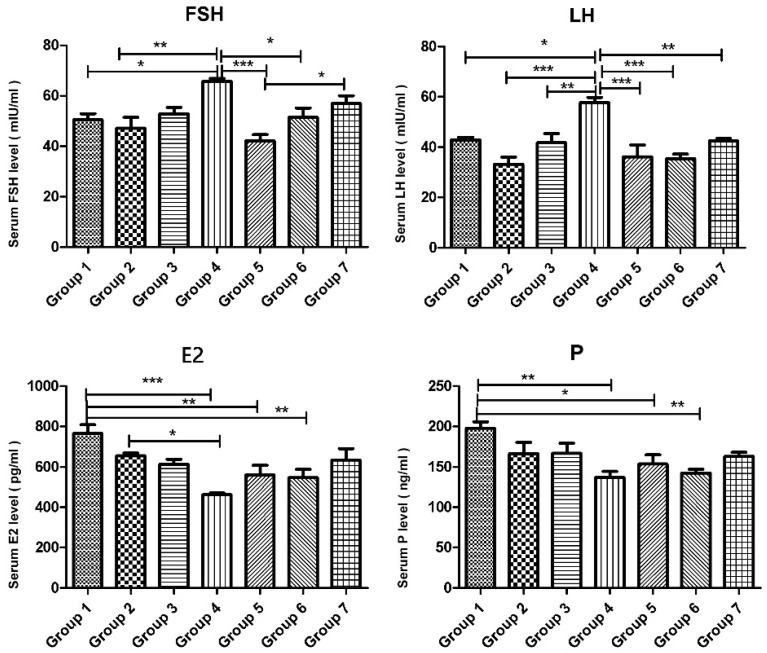
Serum levels of FSH (mIU/mL), LH (mIU/mL), E2 (pg/mL), and P (ng/mL) of the experimental groups. Data are presented as mean ± standard deviation (SD). * *p* < 0.05, ** *p* < 0.01, *** *p* < 0.001.

FSH levels were significantly increased in the cyclophosphamide group compared to the control groups, whereas they were markedly reduced in groups 5, 6, and 7 compared to the cyclophosphamide group (*p* < 0.0001). Similarly, LH levels were significantly higher in the cyclophosphamide group compared to groups 1, 2, and 3. However, in the treatment groups (groups 5, 6, and 7), LH levels were comparable to those in the control groups (*p* < 0.0001, *p* = 0.0026). E2 levels in the intact control group were significantly higher than in the cyclophosphamide, cyclophosphamide + melatonin, and cyclophosphamide + vitamin D3 groups. However, in the group treated with cyclophosphamide, melatonin, and vitamin D, E2 levels were similar to those in group 1 (*p* = 0.0008). Progesterone levels were significantly decreased in groups 4, 5, and 6 compared to group 1, and this reduction was statistically significant (*p* = 0.018). However, no statistically significant difference was observed between the control group and group 7 in terms of P levels (Figure 1).

### 2.2. Light Microscopic Findings

**Figure 2:**
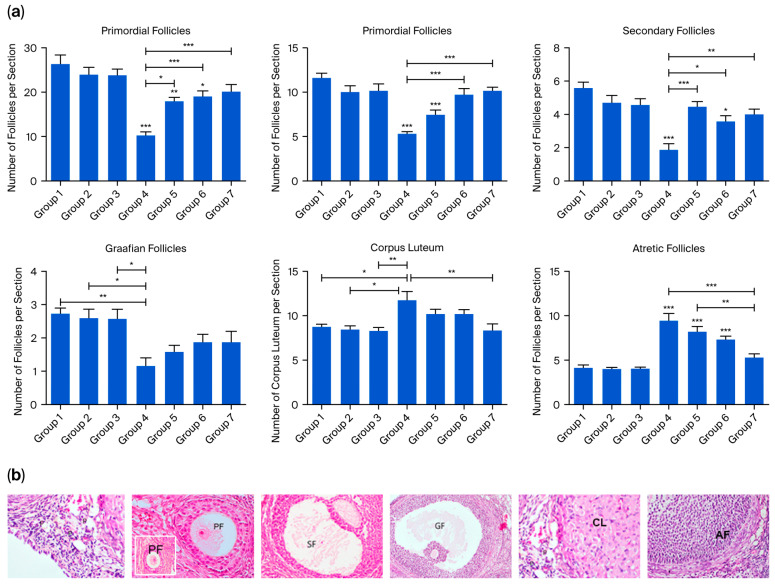
(**a**) Quantitative analysis of the number of ovarian follicle counts, including primordial, primary, secondary, Graafian, and atretic follicles, as well as corpus luteum. Data are presented as mean ± standard deviation (SD). * *p* < 0.01, ** *p* < 0.001, *** *p* < 0.0001. (**b**) Representative ovarian sections used for follicle counting. Primordial follicles (p) were defined as small, quiescent primary oocytes surrounded by a single layer of flattened granulosa cells. Primary follicles (PF) were characterized by a primary oocyte enclosed by a single or multiple layers of cuboidal granulosa cells. Follicles with a visible antrum between granulosa cells were classified as secondary follicles (SF), while large follicles with a fully developed antral cavity were defined as Graafian follicles (GF). Follicles exhibiting degenerative changes, such as pyknotic nuclei in granulosa cells, oocyte fragmentation, or disrupted follicular architecture, were identified as atretic follicles (AF). The corpus luteum (CL) was defined as a post-ovulatory structure composed of large, eosinophilic luteal cells arranged in a lobulated or folded pattern with abundant cytoplasm and central blood vessels, indicative of luteinization.

Ovarian tissue in serial sections stained with H&E was evaluated using a light microscope. The number of primordial, primary, secondary, Graafian, and atretic follicles, as well as corpora lutea, was counted and histologically evaluated. Statistical analysis of follicle counts in the serial sections of the cyclophosphamide-treated group revealed a significant reduction in the number of normal primordial, primary, secondary, and Graafian follicles compared to groups 1, 2, and 3. The decrease in primordial, primary, and secondary follicles was statistically significant (*p* < 0.0001), and the reduction in Graafian follicles was also statistically significant (*p* < 0.01). In groups 5, 6, and 7, the numbers of primordial, primary, and secondary follicles were significantly higher than in group 4 (*p* < 0.0001), whereas the increase in Graafian follicle numbers was not statistically significant. Notably, the number of corpora lutea and atretic follicles was the highest in group 4 compared to all other groups. Comparisons of atretic follicle counts among groups revealed no significant differences among the control groups (groups 1, 2, and 3). However, in groups 4, 5, and 6, follicular atresia was significantly higher than in the control groups (*p* < 0.0001). In contrast, group 7 exhibited a significant reduction in follicular atresia compared to the cyclophosphamide group (*p* < 0.0001) (Figure 2).

**Figure 3:**
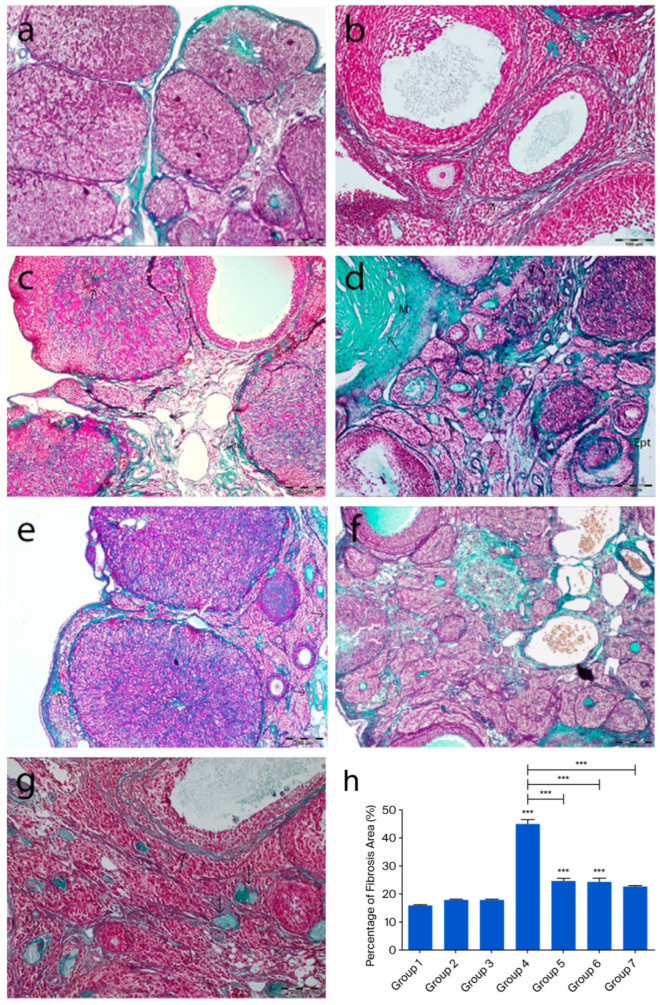
Representative ovarian sections from the following groups are shown: (**a**) group 1—intact control, (**b**) group 2—melatonin, (**c**) group 3—vitamin D3, (**d**) group 4—cyclophosphamide, (**e**) group 5—cyclophosphamide + melatonin, (**f**) group 6—cyclophosphamide + vitamin D3, and (**g**) group 7—cyclophosphamide + melatonin + vitamin D3. Masson’s trichrome staining of ovarian tissue showing green-stained collagen fibers (arrows). (**h**) Quantification of fibrosis using ImageJ, 1.54d, expressed as a percentage of green-stained area relative to total tissue. *** *p* < 0.001. Scale bars: 200 µm (**a**,**c**,**e**,**f**), 100 µm (**b**), 500 µm (**d**,**g**).

Masson’s trichrome-stained ovarian sections from the control groups displayed normal histology, whereas in group 4, a marked increase in collagen fibers was observed in both the cortical and medullary regions. Collagen fiber accumulation was evident in the tunica albuginea, the stroma surrounding atretic follicles, and the connective tissue between luteal cells in some corpora lutea. Additionally, an increase in collagen fibers was noted in the stroma surrounding blood vessels in the medullary region. In the treatment groups (groups 5, 6, and 7), collagen fiber deposition was relatively reduced, and in group 7, the collagen staining pattern was similar to that of the control groups (*p* < 0.0001) (Figure 3).

### 2.3. Molecular Biological Findings

**Figure 4:**
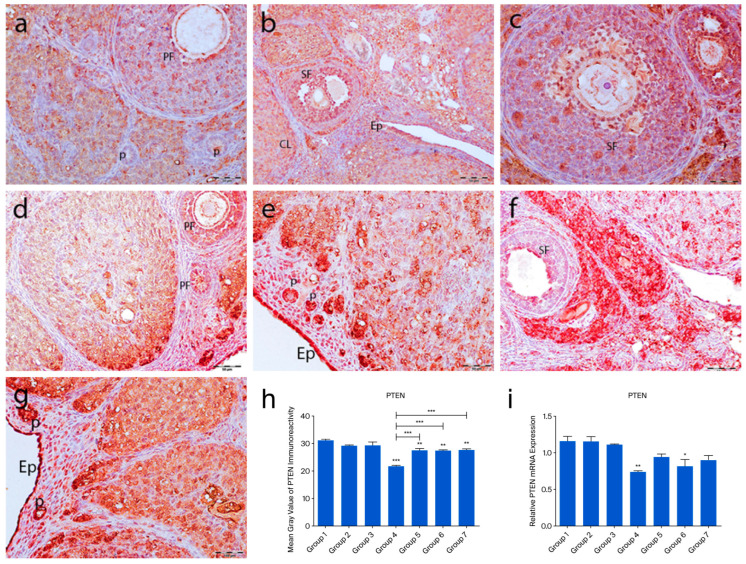
PTEN immunoreactivity and the graphical representation of PTEN immunoreactivity scores in ovarian tissues belonging to the following groups are shown: group 1—intact control (**a**), group 2—melatonin (**b**), group 3—vitamin D3 (**c**), group 4—cyclophosphamide (**d**), group 5—cyclophosphamide and melatonin (**e**), group 6—cyclophosphamide and vitamin D3 (**f**), group 7—cyclophosphamide, melatonin, and vitamin D3 (**g**). Immunohistochemical staining intensity (**h**) and mRNA expression profiles of PTEN in the ovarian tissues of the experimental groups (**i**). Data are expressed as mean ± standard deviation (SD). * *p* < 0.05, ** *p* < 0.01, *** *p* < 0.001. Epithelium (Ep), primordial follicle (p), primary follicle (PF), secondary follicle (SF), corpus luteum (CL). Scale bars: 50 µm (**a**–**f**), 100 µm (**g**).

**Figure 5:**
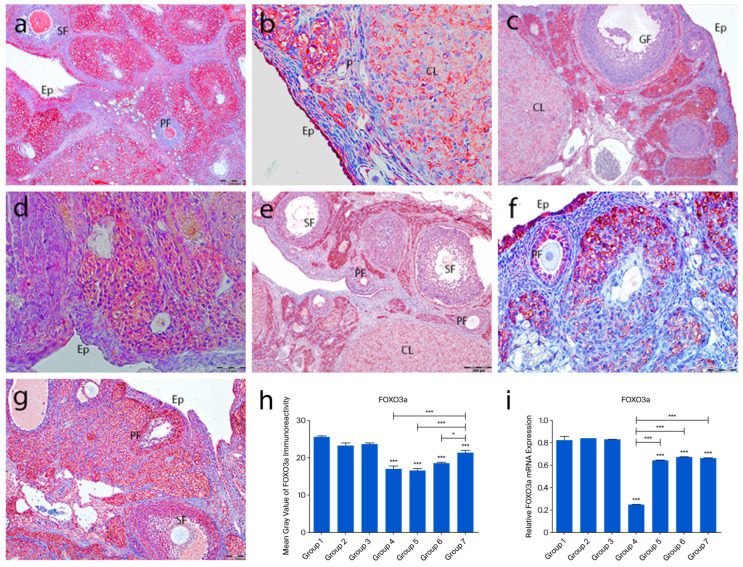
FOXO3a immunoreactivity and the graphical representation of FOXO3a immunoreactivity scores in ovarian tissues of the following groups are shown: group 1—intact control (**a**), group 2—melatonin (**b**), group 3—vitamin D3 (**c**), group 4—cyclophosphamide (**d**), group 5—cyclophosphamide and melatonin (**e**), group 6—cyclophosphamide and vitamin D3 (**f**), and group 7—cyclophosphamide, melatonin, and vitamin D3 (**g**). Immunohistochemical staining intensity (**h**) and mRNA expression profiles of FOXO3a in the ovarian tissues of the experimental groups (**i**). Data are expressed as mean ± standard deviation (SD). * *p* < 0.05, *** *p* < 0.001. Observed structures include epithelium (Ep); primordial (p), primary (PF), secondary (SF), Graafian (GF), and corpus luteum (CL). Scale bars: 100 µm (**a**,**c**,**f**), 50 µm (**b**), 200 µm (**d**,**e**,**g**).

**Figure 6:**
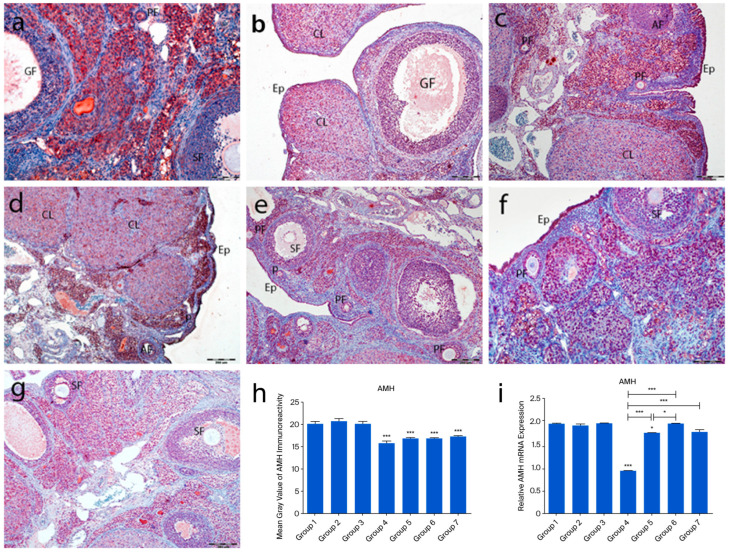
AMH immunoreactivity and the graphical representation of AMH immunoreactivity scores in ovarian tissues of the following groups are shown: group 1—intact control (**a**), group 2—melatonin (**b**), group 3—vitamin D3 (**c**), group 4—cyclophosphamide (**d**), group 5—cyclophosphamide and melatonin (**e**), group 6—cyclophosphamide and vitamin D3 (**f**), and group 7—cyclophosphamide, melatonin, and vitamin D3 (**g**). Immunohistochemical staining intensity (**h**) and mRNA expression profiles of AMH in the ovarian tissues of the experimental groups (**i**). Data are expressed as mean ± standard deviation (SD). * *p* < 0.05, *** *p* < 0.001. Observed structures include epithelium (Ep) and primordial (p), primary (PF), secondary (SF), Graafian (GF), and atretic follicles. Scale bars: 100 µm (**a**,**f**), 200 µm (**b**–**e**,**g**).

The expression profiles of PTEN, FOXO3a, and AMH mRNAs in rat ovarian tissue were analyzed using RT-PCR. After cyclophosphamide-induced ovarian damage, the expressions of FOXO3a, AMH, and PTEN were found to be decreased compared to the control gene (GAPDH). No significant difference was observed in PTEN expression among the control groups. However, when all three control groups were compared to group 4, a significant reduction in PTEN expression was detected in group 4 (*p* = 0.006). In group 6, a significant decrease was observed compared to groups 1 and 3 (*p* < 0.01), while in group 5, a significant reduction was noted compared to group 2 (*p* < 0.01) (Figure 4i). FOXO3a expression did not show significant differences among groups 1, 2, and 3. However, in groups 4, 5, 6, and 7, FOXO3a expression was significantly reduced compared to the control group (*p* < 0.0001). Notably, when the FOXO3a expression levels in groups 5, 6, and 7 were compared to those in group 4, a significant increase was observed (*p* < 0.0001) (Figure 5i). Regarding AMH expression, no significant differences were found among the control groups. In group 4, a significant reduction in AMH levels was detected in group 4 (*p* < 0.0001). Additionally, AMH levels in groups 5, 6, and 7 were significantly higher compared to those in group 4 (*p* < 0.0001). Notably, AMH levels in group 5 were lower than those in groups 1, 3, and 6 (*p* < 0.01) (Figure 6i).

### 2.4. Immunohistochemical Findings

#### 2.4.1. PTEN Immunoreactivity

In the control groups (1–3), PTEN staining was absent in primordial follicle oocytes, strong in granulosa cells of developing follicles, and moderate in corpora lutea and interstitial gland cells (Figure 4a–c). In the cyclophosphamide group (group 4), weak staining appeared in primordial follicle oocytes and moderate staining in developing follicles and granulosa cells. Some corpora lutea showed partial or no staining (Figure 4d). In the treatment groups (5–7), a mix of stained and unstained primordial follicles was observed. Primary oocytes were mostly negative, granulosa cells showed weak cytoplasmic staining, and interstitial gland cells displayed strong immunoreactivity (Figure 5e–g). Quantitative analysis revealed significantly reduced PTEN expression in group 4 compared to controls (*p* < 0.0001). Groups 5–7 also showed lower expression than group 1 (*p* = 0.0183), but significantly higher levels than group 4 (*p* < 0.0001) (Figure 4h).

#### 2.4.2. FOXO3a Immunoreactivity

FOXO3a immunoreactivity was detected in the nuclei of ovarian surface epithelial cells but not in the tunica albuginea. In the control groups (1–3), strong nuclear staining was observed in oocytes of primordial, primary, and secondary follicles. Granulosa cells showed weak cytoplasmic staining, and theca cells were mostly negative. In the POF group (group 4), nuclear staining in oocytes and follicular cells was lost, with only moderate cytoplasmic staining in oocytes. Granulosa cells showed strong cytoplasmic staining, while theca layers remained unstained. In the treatment groups (5–7), FOXO3a staining patterns resembled those of the controls, with strong oocyte staining and weak granulosa/theca staining. Quantitative analysis showed a significant decrease in FOXO3a expression in group 4 compared to the controls (*p* < 0.0001). Groups 5–7 showed increased expression compared to group 4, with group 7 showing the most prominent recovery (*p* < 0.0001) (Figure 5a–h).

#### 2.4.3. AMH Immunoreactivity

In the control groups (1–3), strong AMH immunoreactivity was observed in granulosa and theca cells of primary and secondary follicles, while Graafian follicles showed weak staining. AMH expression was absent in primordial follicles and theca cells of atretic follicles. Cumulus cells in Graafian follicles exhibited low staining, and corpora lutea showed variable immunoreactivity (Figure 6a–c). In group 4, some primordial follicle oocytes showed AMH staining, while others did not. Strong staining was noted in interstitial gland cells, and moderate staining in corpora lutea (Figure 6d). In the treatment groups (5–7), primordial follicles lacked AMH expression, but granulosa cells of primary and secondary follicles showed strong staining. Graafian follicles exhibited weak staining (Figure 6e–g). Quantitative analysis revealed significantly reduced AMH immunoreactivity in groups 4–7 compared to controls (*p* < 0.0001). Although AMH levels were higher in groups 5–7 than in group 4, the difference was not statistically significant (*p* = 0.2718) (Figure 6h).

#### 2.4.4. Active Caspase-3 Immunoreactivity

**Figure 7:**
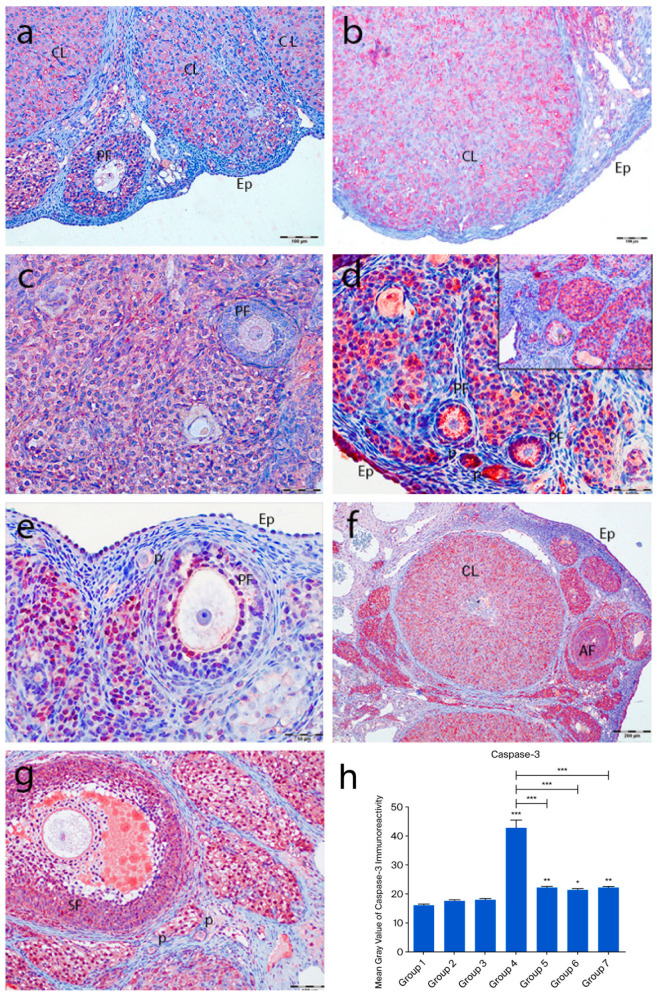
Active caspase-3 immunoreactivity and the graphical representation of immunoreactivity scores in ovarian tissues of the following groups are shown: group 1—intact control (**a**), group 2—melatonin (**b**), group 3—vitamin D3 (**c**), group 4—cyclophosphamide (**d**, **d** inset), group 5—cyclophosphamide and melatonin (**e**), group 6—cyclophosphamide and vitamin D3 (**f**), and group 7—cyclophosphamide, melatonin, and vitamin D3 (**g**). The staining intensity was quantified using the mean gray value in ImageJ 1.54d (**h**) (mean ± SD, *p* < 0.05). * *p* < 0.05, ** *p* < 0.01, *** *p* < 0.001. Observed structures include epithelium (Ep); primordial (p), primary (PF), secondary (SF), and atretic follicles (AF); and corpus luteum (CL). Scale bars: 100 µm (**a**,**f**,**g**), 50 µm (**b**–**e**).

In the control groups (1–3), no active caspase-3 staining was detected in primordial follicles, follicular cells, or granulosa cells. However, moderate staining was observed in corpora lutea and interstitial gland cells (Figure 7a–c). In the cyclophosphamide group (group 4), strong immunoreactivity was seen in oocytes and follicular cells of primordial follicles (Figure 7d, inset), with moderate staining in primary, secondary, and Graafian follicle oocytes. Granulosa cells showed intense staining, and regressing corpora lutea exhibited increased caspase-3 expression (Figure 7d). In the treatment groups (5–7), some primordial follicles lacked staining, while others showed moderate immunoreactivity. Developing follicle oocytes displayed moderate staining, and granulosa cells exhibited variable intensity (Figure 7e–g). Quantitative analysis showed a significant increase in caspase-3 immunoreactivity in group 4 compared to controls (*p* < 0.0001), while groups 5–7 showed significantly lower levels than group 4 (*p* < 0.0001) (Figure 7h).

#### 2.4.5. TNF-α Immunoreactivity

**Figure 8:**
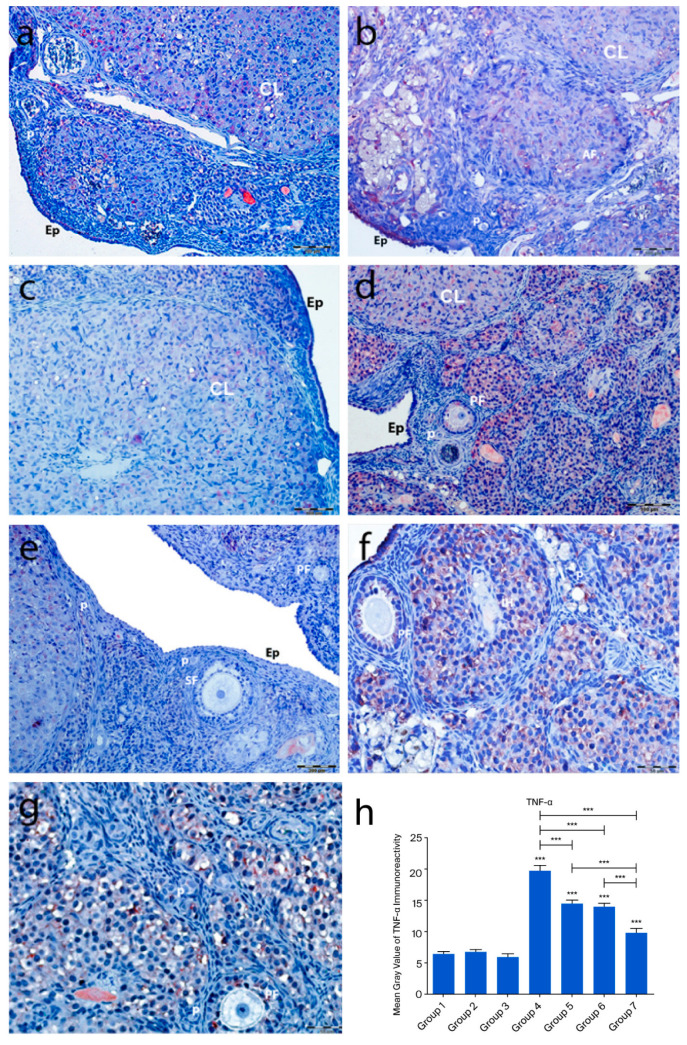
TNF-α immunoreactivity and the graphical representation of TNF-α immunoreactivity scores in ovarian tissues of the following groups are shown: group 1—intact control (**a**), Group 2—melatonin (**b**), group 3—vitamin D3 (**c**), group 4—cyclophosphamide (**d**), group 5—cyclophosphamide and melatonin (**e**), group 6—cyclophosphamide and vitamin D3 (**f**), and group 7—cyclophosphamide, melatonin, and vitamin D3 (**g**). The staining intensity was quantified using the mean gray value in ImageJ 1.534d (**h**) (mean ± SD, *p* < 0.05). *** *p* < 0.001. Observed structures include epithelium (Ep), primordial follicles (p), primary follicles (PF), atretic follicles (AF), and corpus luteum (CL). Scale bars: 200 µm (**a**,**d**,**e**), 50 µm (**b**,**c**,**f**,**g**).

In the control groups (1–3), TNF-α immunoreactivity was weak, with no staining in primordial follicle oocytes, follicular cells, or granulosa cells. Moderate to strong staining was observed in atretic follicles, corpora lutea, and interstitial gland cells (Figure 8a–c). In the cyclophosphamide group (group 4), moderate TNF-α staining appeared in primordial and developing follicle oocytes and follicular cells. Granulosa cells and luteal cells showed intense staining, particularly in regressing corpora lutea and macrophages (Figure 8d). In the treatment groups (5–7), TNF-α expression in primordial follicles ranged from absent to moderate. Developing follicles showed moderate staining, while granulosa and luteal cells exhibited variable intensity (Figure 8e–g). No significant differences were found among control groups. However, TNF-α immunoreactivity was significantly increased in group 4 compared to controls (*p* < 0.0001). Groups 5–7 showed markedly reduced TNF-α expression compared to group 4 (*p* < 0.0001), with group 7 showing the greatest reduction (*p* < 0.001), approaching control levels (Figure 8h).

### 2.5. Electron Microscopic Findings

**Figure 9:**
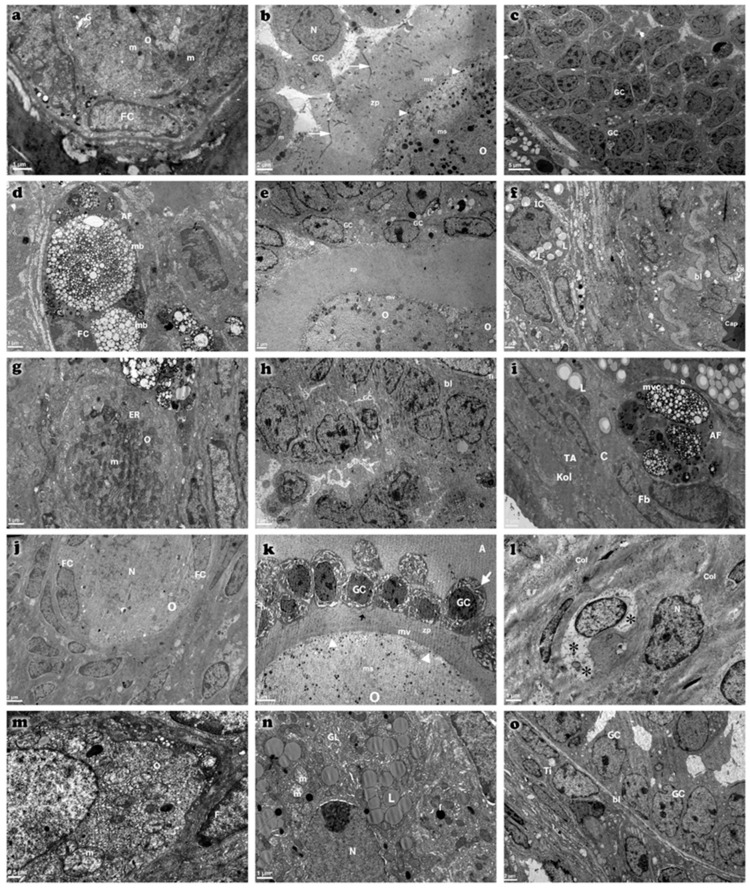
Transmission electron micrographs of ovarian tissues from control (**a**–**c**), cyclophosphamide (**d**–**f**), melatonin-treated (**g**–**i**), vitamin D3-treated (**j**–**l**), and combined treatment (**m**–**o**) groups. Observed ultrastructural components include oocyte (O), granulosa cells (GC), follicular cells (FC), theca interna (Ti), and capillary (Cap). Atretic primordial follicles (AF) are evident, characterized by nuclear pyknosis in granulosa cells and marked dilation of cytoplasmic organelles (indicated by arrows). Microvilli (mv), tunica albuginea (TA), and basal lamina (bl) are clearly distinguished. Multivesicular bodies (mb), membranous structures (ms), cortical granules (arrowhead), and zona pellucida (zp) are also identified. Ovarian stromal cells exhibit lytic areas (*) in their cytoplasm. Additional structures include spherical nuclei (N), mitochondria (m), and lipid droplets (L). Scale bar: 5 µm (**c**,**k**), 2 µm (**b**,**e**,**h**,**j**,**o**), 1 µm (**a**,**d**,**f**,**g**,**i**,**l**,**n**), 0.5 µm (**m**).

Electron microscopic examinations revealed increased collagen fiber deposition in the tunica albuginea, thickening of the basal lamina of blood vessels in the stroma, and degenerative changes in primordial follicle oocytes in the cyclophosphamide-treated group. These changes included increased heterochromatin granules in the nucleus, cytoplasmic vacuolization, loss of cytoplasmic organelles, and an increase in lysosomes and multivesicular bodies. Additionally, developing follicle oocytes exhibited segmentation, thickening and irregularities in the zona pellucida, and a reduction in microvilli on the oocyte surface. Atretic changes were characterized by the presence of apoptotic bodies between fragmented oocytes and granulosa cells. Although atretic changes were still present in the treatment groups, their severity was reduced compared to the cyclophosphamide group (Figure 9).

## 3. Discussion

In the present study, cyclophosphamide administration significantly affected the primordial follicle reserve in rat ovaries. This pathological process resulted in the recruitment of a large number of primordial follicles into the growth phase and the subsequent atresia of developing follicles, leading to a marked increase in the total number of atretic follicles. In the cyclophosphamide-treated group, serum FSH and LH levels increased significantly, whereas E2 and P levels declined markedly. These biochemical findings confirm the development of POF after a single intraperitoneal dose of 100 mg/kg cyclophosphamide [11].

The levels of PTEN, FOXO3a, and AMH, which are known to exert inhibitory effects during follicular development, were significantly altered by cyclophosphamide exposure. However, co-treatment with melatonin and vitamin D3 helped maintain their expression levels, contributing to the preservation of the ovarian reserve. Histological assessment revealed a pronounced reduction in the numbers of morphologically normal primordial, primary, secondary, and Graafian follicles in the cyclophosphamide group, accompanied by increased corpora lutea and atretic follicles. In the treatment groups, however, the number of primordial, primary, and secondary follicles displaying normal histological features rose significantly. Because the size of the primordial follicle pool defines the ovarian reserve, toxic stimuli that specifically target primordial follicles are known to shorten the reproductive lifespan and precipitate POF [12].

Cyclophosphamide-induced ovarian damage was associated with a reduction in the mRNA expression levels of the PTEN, FOXO3a, and AMH genes, which are known to inhibit follicular activation and further follicular development [13]. In contrast, the expression levels of these genes were increased in the treatment groups, showing values comparable to those in the control groups. Immunohistochemical findings supported the molecular data, revealing a positive correlation between gene expression and protein localization. The PI3K/PTEN/AKT signaling pathway plays a pivotal role in regulating the transition of primordial follicles into the active growth phase. In this pathway, PTEN and FOXO3a function as key negative regulators. Cyclophosphamide exposure led to a marked reduction in both mRNA and protein levels of PTEN and FOXO3a, suggesting abnormal activation of this signaling cascade. Previous studies have demonstrated that anticancer drugs can activate the PI3K/AKT pathway, thereby promoting mass activation of dormant primordial follicles [13,14]. Within this context, AKT activation suppresses p27, a cell cycle inhibitor, thereby facilitating the transition of primordial follicles to the primary stage [15]. Albamonte et al. reported that in patients diagnosed with extragonadal cancers who underwent ovarian cryopreservation, approximately 50% of primordial follicle oocytes exhibited PTEN expression regardless of chemotherapy exposure; however, PTEN expression was significantly reduced in patients who received chemotherapy [16]. FOXO3a plays a critical role in cellular homeostasis by regulating the expression of genes involved in DNA repair, cell cycle control, oxidative stress response, redox signaling, gluconeogenesis, and apoptosis [17]. Furthermore, FOXO3a has been reported to be highly expressed in the oocyte nucleus of primordial follicles, where it acts as a determinant of ovarian reserve [13,17]. In our immunohistochemical analysis, FOXO3a expression was predominantly negative in the oocyte nuclei and follicular cells of primordial follicles in the cyclophosphamide group, whereas moderate cytoplasmic staining was observed in the ooplasm. In rodents, FOXO3a transcriptional activity suppresses follicular growth when localized in the oocyte nucleus, while its phosphorylation-induced translocation to the cytoplasm triggers follicular activation [18]. In our study, the frequent cytoplasmic localization of FOXO3a in oocytes of primordial follicles suggested that these follicles had entered the activation phase. Considering the findings by Castrillon et al., who demonstrated that FOXO3a knockout mice exhibited massive follicular activation leading to early depletion of functional follicles and infertility, it was concluded that FOXO3a-negative follicles or those exhibiting cytoplasmic expression are likely undergoing rapid activation and entry into the follicular growth trajectory. Similarly, the mRNA expression and immunostaining intensity of AMH, a negative regulator secreted by granulosa cells that inhibits follicular growth, were found to be markedly reduced in the cyclophosphamide-treated group. Our light and electron microscopic observations revealed that a large proportion of follicles entering the growth phase underwent atresia and that granulosa cells exhibited advanced degenerative changes. These findings support the notion that the reduced AMH production in these follicles is a consequence of granulosa cell dysfunction.

When evaluated individually, both melatonin and vitamin D3 have been demonstrated to exert partial but distinct protective effects against cyclophosphamide-induced ovarian damage. Melatonin, through its antioxidant and anti-apoptotic properties, was shown to decrease FSH and LH levels while increasing serum E2 and progesterone concentrations. This hormonal modulation contributed to the attenuation of atresia in follicles that had entered the growth phase. Furthermore, the observed upregulation of PTEN, FOXO3a, and AMH at both gene and protein levels suggests that melatonin may contribute to the relative preservation of the primordial follicle pool, possibly by inhibiting premature follicular activation. In contrast, vitamin D3 exhibited a different mechanism of action. In addition to its antioxidant effects, it modulated the expression of key regulatory genes involved in folliculogenesis. The increase in PTEN, FOXO3a, and AMH expression, accompanied by a higher number of morphologically normal follicles and reduced levels of fibrosis, indicates that vitamin D3 may enhance follicular viability and structural integrity. Moreover, the elevated serum E2 and progesterone concentrations observed in the vitamin D3-treated group reflect a functional improvement in steroidogenic capacity. Nevertheless, although both agents showed beneficial effects independently, a more pronounced improvement in ovarian structure and function was observed in the combined treatment group. This finding points toward a potential synergistic interaction between melatonin and vitamin D3, rather than a merely additive effect.

In the group receiving combined treatment with melatonin and vitamin D3, the expression levels of PTEN, FOXO3a, and AMH were maintained at levels comparable to those of the control group, highlighting the protective effect of this combination. Melatonin and vitamin D3 are generally recognized as key regulators of circadian and metabolic homeostasis, and they share common properties, including antioxidant, anti-inflammatory, and mitochondrial modulatory effects [19]. While the combined effects of these agents on ovarian damage have not been extensively studied in morphological analyses, increasing evidence suggests a synergistic potential between these two molecules [19,20,21]. For instance, melatonin has been shown to regulate vitamin D metabolism via nuclear receptors, and their combined use has been reported to reduce cardiotoxicity and improve oocyte and embryo quality in IVF applications [22,23,24]. In our study, cyclophosphamide-induced disruption of follicular structure and steroidogenesis was partially ameliorated by the co-administration of melatonin and vitamin D3, which helped preserve follicular integrity and reduce fibrosis. Additionally, the combination therapy group exhibited an increase in the number of morphologically normal follicles and a decrease in atretic follicles. Taken together, these findings suggest that the co-treatment with melatonin and vitamin D3 may restore ovarian function in the context of cyclophosphamide-induced damage, possibly through the modulation of PTEN, FOXO3a, and AMH at both gene and protein levels. Although vitamin D3 and melatonin share antioxidant properties, they also act through distinct receptor-mediated signaling pathways [25]. To delineate this difference more precisely, advanced pathway-specific analyses are required. Future studies employing receptor antagonists, transcriptomic profiling, or pathway-specific biomarkers may help determine whether the observed protective effects are primarily receptor-driven or attributable to the suppression of oxidative stress. The absence of a control group treated with the combination of melatonin and vitamin D3 without POF induction limits the ability to assess the baseline physiological effects of this treatment. Inclusion of such a group in future studies may offer a better understanding of the potential therapeutic effects under non-pathological conditions.

In the cyclophosphamide group, the expression levels of active caspase-3 and TNF-α were markedly elevated, whereas these levels were significantly reduced in the treatment groups. In addition to apoptosis, other programmed cell death pathways, such as necroptosis, also contribute to follicular atresia [26]. Electron microscopic analyses revealed ultrastructural changes characteristic of necroptosis in granulosa cells, including organelle swelling, increased lysosomal content, and disruption of plasma membrane integrity. The high TNF-α immunoreactivity observed in the cyclophosphamide group supports the notion that an inflammation-mediated necroptotic process may have been activated. Although immunohistochemistry and qRT-PCR analyses provided important insights into the expression patterns of PTEN, FOXO3a, AMH, caspase-3, and TNF-α, Western blot analysis was not feasible due to protocol constraints and insufficient tissue availability. Future studies will prioritize the inclusion of Western blotting to quantitatively validate these key molecular targets and further support the mechanistic conclusions drawn. Although the protective effects of the combination therapy were demonstrated at hormonal and histological levels, the present study did not conduct an in-depth evaluation of molecular pathways such as apoptosis, oxidative stress, and inflammation. This represents a key limitation. Future investigations should aim to include pathway-specific markers (BAX, Bcl-2, 8-OHdG, SOD, MDA, IL-1β) to better elucidate the mechanistic basis of the observed therapeutic effects [27]. In primordial follicles, increased multivesicular body formation and accumulation of nuclear heterochromatin were noted, while in developing follicles, oocyte segmentation and apoptotic changes in granulosa cells were observed. These findings indicate that follicular atresia occurs not only through apoptosis but also involves TNF-α-mediated necroptosis and ultrastructural dysfunction in oocytes and granulosa cells, suggesting the involvement of multiple cell death pathways in this process. At the electron microscopic level, increased formation of multivesicular bodies, accumulation of heterochromatin, and organelle degeneration reveal the complex biological responses of cells to stress and support the notion that chemotherapy-induced ovarian toxicity is shaped by a multifaceted pathogenesis. Active caspase-3 staining showed the highest immunoreactivity in the cyclophosphamide group, confirming the presence of enhanced apoptotic activity. Electron microscopy also revealed features consistent with oxidative stress-related damage and fibrosis, such as collagen accumulation, thickening of vascular basement membranes, and oocyte degeneration. These morphological alterations are likely to impair follicular vascularization and nutrient diffusion, thereby accelerating follicular atresia. However, in the treatment groups, such pathological alterations were significantly reduced, and the ultrastructural features closely resembled those of the control group.

Although the combination of melatonin and vitamin D3 did not show statistically significant superiority over monotherapies across all parameters, certain findings such as better preservation of follicular structure and a more balanced hormonal profile indicate biologically meaningful advantages. In the combination group, follicular atresia was reduced, antral follicles were preserved, and endocrine function was partially restored. These effects are thought to result from the complementary actions of melatonin on mitochondrial oxidative stress and vitamin D3 on immuno-inflammatory responses. Despite limited statistical differences, the combination therapy was concluded to offer broader cytoprotective potential. Cyclophosphamide administration significantly reduced the ovarian reserve by inducing widespread activation of primordial follicles followed by atresia. The combination of melatonin and vitamin D3 alleviated these effects by preserving follicular integrity and partially restoring hormonal balance. While the current study did not directly investigate alternative cell death pathways, such as necroptosis or pyroptosis, these mechanisms may represent valuable targets for future mechanistic studies. Further research is also needed to define optimal treatment protocols and assess the translational potential of this combination therapy in fertility preservation contexts.

Taken together, these data suggest that cyclophosphamide-induced ovarian damage is not limited to apoptosis alone, but rather encompasses a complex and multilayered pathological process involving oxidative stress, inflammation, fibrosis, and necroptosis. The combined administration of melatonin and vitamin D3 not only modulated molecular markers but also preserved follicular architecture, vascular integrity, and ultrastructural organization, thereby exerting a comprehensive restorative and homeostatic effect at both the cellular and tissue levels. This synergistic approach holds significant translational potential as a promising fertility preservation strategy for individuals exposed to gonadotoxic therapies.

## 4. Materials and Methods

### 4.1. Acquisition of Experimental Animals and Housing Conditions

All procedures were conducted in accordance with the 1986 Universal Declaration of Animal Rights in Strasbourg and were performed under veterinary supervision with the approval of the ethics committee. Prior to the experiment, approval was obtained from the Çukurova University Animal Experiments Local Ethics Committee on 8 July 2021 (Meeting No: 6). A total of 49 female Wistar albino rats, aged 7–8 weeks and weighing 200–250 g, were obtained from the Çukurova University SABİDAM unit. Female rats were isolated from male rats for 21 days. All animals were provided with food and water ad libitum. The ambient temperature was maintained at 22 ± 2 °C, and a 12 h light/12 h dark cycle was applied.

### 4.2. Experimental Design and Procedures

**Figure 10:**
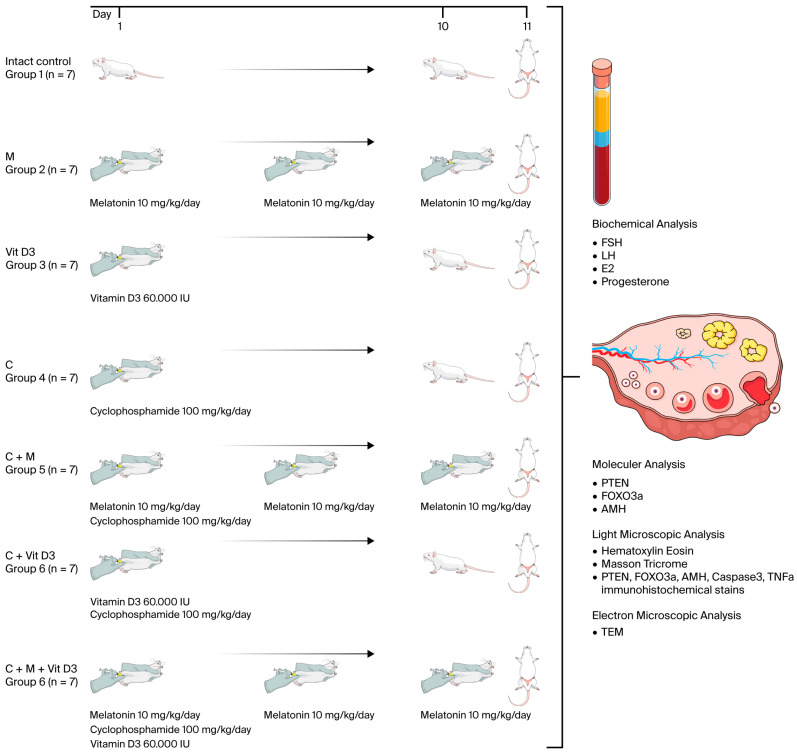
Experimental design and procedures. The illustrations used in this figure were obtained from Mind the Graph (www.mindthegraph.com) (accessed on 11 July 2025) under the appropriate license.

Adult female rats (*n* = 49) were randomly assigned to seven groups (*n* = 7 per group). In the study, no treatment was administered to group 1 (intact control), while group 2 received melatonin alone, group 3 received vitamin D3 alone, group 4 received cyclophosphamide alone, group 5 received a combination of melatonin and cyclophosphamide, group 6 received vitamin D3 and cyclophosphamide, and group 7 received a combination of melatonin, vitamin D3, and cyclophosphamide. On day 1, animals received a single intraperitoneal (i.p.) injection of cyclophosphamide and/or vitamin D3 according to their respective group protocols. Subsequently, melatonin was administered daily via i.p. injection for 10 consecutive days in the relevant groups. A single dose of 100 mg/kg cyclophosphamide (Endoxan Baxter Healthcare Corporation, Deerfield, IL, USA) was administered to induce a model of premature ovarian failure. Melatonin (Cayman Chemical Company, Ann Arbor, MI, USA; Cat. No: 14427) and vitamin D (Ostriol, 1 mcg/mL, VEM İlaç Sanayi ve Ticaret A.Ş., İstanbul, Turkey) were used as fertoprotective agents. At the end of the experimental period (day 11), all animals were euthanized via an intraperitoneal injection of xylazine (10 mg/kg) and ketamine (80 mg/kg). Blood samples were obtained for biochemical assessment, and ovarian tissues were excised for subsequent molecular, histological, and immunohistochemical analyses. The experimental design and procedures are presented in Figure 10.

### 4.3. Biochemical Analyses

Intracardiac blood samples were collected under anesthesia and centrifuged at 3000 rpm for 10 min to obtain serum. The separated serum samples were aliquoted and stored at −80 °C until analysis. Serum levels of FSH, LH, E2, and progesterone (P) were quantitatively determined using rat-specific commercial ELISA kits (Sunred Biological Technology Co., Ltd., Shanghai, China). The catalog numbers were as follows: FSH (Cat. No. 201-11-0183), LH (Cat. No. 201-11-0180), estradiol (Cat. No. 201-11-0175), and progesterone (Cat. No. 2011-10-742). All measurements were performed in triplicate according to the manufacturer’s instructions, and absorbance was read at 450 nm using a microplate reader.

### 4.4. Light Microscopy Tissue Preparation

One ovary from each rat was fixed in 10% neutral formalin for light microscopic analysis and processed using a Leica automatic tissue processor. Serial sections were obtained from paraffin-embedded tissue blocks at 50 µm intervals, and histological staining was performed using hematoxylin and eosin (H&E), Masson’s trichrome, and immunohistochemical techniques [28]. The stained sections were examined and photographed using an Olympus BX53 (Olympus Corporation, Tokyo, Japan) light microscope. Ovarian follicles, including primordial, primary, secondary, Graafian, atretic follicles, and corpus luteum, were histologically evaluated. Follicles were classified as follows: primordial follicles, consisting of a primary oocyte surrounded by 2–3 flattened follicular cells; primary follicles, composed of a primary oocyte and a single or multiple layers of cuboidal granulosa cells; secondary follicles, identified by the presence of an antrum between the primary oocyte and surrounding granulosa cells; graafian follicles, large follicles with a fully developed antrum; atretic follicles, follicles exhibiting degenerative changes in the oocyte and granulosa cells [29]. Follicle counts were performed in serial sections taken at 50 µm intervals.

For Masson trichrome staining, paraffin sections were incubated in Bouin’s solution at 60 °C for 1 h, deparaffinized, and stained sequentially with iron hematoxylin, Ponceau xylidine–acid fuchsin–glacial acetic acid solution (Solution A), phosphomolybdic acid, and light green SF yellowish glacial acetic acid (Solution C) [30]. The stained sections were analyzed under a light microscope, and images were captured. The percentage of stained area was quantitatively assessed using ImageJ 1.54d software.

### 4.5. Immunohistochemical Analysis

To assess the expression levels of PTEN, FOXO3a, AMH, TNF-α, and caspase-3, 5 µm thick sections were mounted on poly-L-lysine-coated slides and deparaffinized at 60 °C. Sections underwent antigen retrieval in a citrate buffer at 95 °C for 30 min. After blocking with 3% hydrogen peroxide (H_2_O_2_) and a protein blocking solution, primary antibodies were applied overnight at 4 °C in a humidified chamber: anti-TNF-α (ABCAM ab220210, Cambridge, UK, 1:200 dilution), anti-caspase-3 (ABCAM ab4051, 1:500 dilution), anti-AMH (Elabscience-AB-15915, Houston, TX, USA, 1:500 dilution), anti-PTEN (Elabscience-AB-93033, 1:100 dilution), and anti-FOXO3a (Elabscience-AB31469, 1:50 dilution). Immunohistochemical staining was visualized the next day using a basic AEC detection kit AEC Detection Kit (Vector Laboratories, Newark, CA, USA), which consisted of an antibody, biotin, streptavidin, and AEC chromogen. After counterstaining with hematoxylin, the tissues were mounted with a coverslip after applying a mounting medium. They were then examined and photographed using an Olympus BX53 light microscope (Olympus Corporation, Tokyo, Japan) To investigate the expressions of PTEN, FOXO3a, AMH, TNF-α, and caspase-3, the average color area percentage of immunostaining was analyzed using the ImageJ 1.54d software.

### 4.6. Electron Microscopy Analysis

For electron microscopy, ovarian tissues were cut into 1 mm^3^ pieces and fixed in 5% glutaraldehyde for 4 h, followed by post-fixation in 1% osmium tetroxide at 4 °C for 2 h. After dehydration, tissues were embedded in polyethylene BEEM capsules (size 00) (Electron Microscopy Sciences, Hatfield, PA, USA). Semi-thin sections were obtained using a Reichert FCS Ultracut S Cryo Ultramicrotome (Depew, NY, USA), and the regions of interest were identified. Ultra-thin (50 nm) sections were stained with uranyl acetate and Reynold’s lead citrate solutions for contrast enhancement. The samples were examined under a JEOL JEM 1400 (JEOL Ltd., Tokyo, Japan) transmission electron microscope, and micrographs were obtained.

### 4.7. Gene Expression Analysis

Total RNA was extracted from ovarian tissues using the PureLink Mini RNA Kit (Invitrogen™, Thermo Fisher Scientific Inc., Waltham, MA, USA; Cat. No: 12183018A) following the manufacturer’s protocol. RNA concentration and purity were determined spectrophotometrically using the A260/A280 ratio, and samples with a ratio ≥ 1.5 were used for further analysis. RNA concentrations were normalized to 100 ng using RNase-free water. cDNA was synthesized from 10 µL of total RNA using the RevertAid First Strand cDNA Synthesis Kit ((Thermo Fisher Scientific Inc., Waltham, MA, USA; Cat. No: K1622) with Oligo(dT)18 primers in a final reaction volume of 20 µL, according to the manufacturer’s instructions. The synthesized cDNA was stored at −80 °C until RT-PCR analysis. RT-qPCR was performed using a SYBR Green kit (Qiagen, Hilden, Germany) on an Applied Biosystems (Thermo Fisher Scientific Inc., Waltham, MA, USA) according to the manufacturer’s instructions. GAPDH was used as the endogenous reference gene for normalization. Relative gene expression levels were calculated using the 2^−ΔΔCt^ method. The gene-specific primer sequences used in the RT-qPCR analyses, including forward and reverse sequences (5′–3′), gene names, and expected amplicon lengths, are detailed in Table 1.

### 4.8. Statistical Analysis

All data were analyzed using GraphPad Prism 5 software (GraphPad Software Inc., USA). The distribution of the data was evaluated using the D’Agostino–Pearson omnibus normality test. For gene expression analysis, relative expression levels were calculated using the 2^−ΔΔCt^ method, representing fold regulation compared to controls.

Statistical comparisons of gene expression among the experimental, treatment, and control groups were performed using one-way analysis of variance (ANOVA), followed by Dunnett’s multiple comparison test for post hoc evaluation. In some cases, Student’s *t*-test was also used to compare selected group pairs. For biochemical, histochemical, immunohistochemical, and follicle count data, one-way ANOVA followed by Bonferroni’s multiple comparison test was used to assess statistical significance among groups. A *p*-value of <0.05 was considered statistically significant in all analyses.

## 5. Conclusions

Cyclophosphamide administration significantly reduces the ovarian reserve by inducing the global activation of primordial follicles and subsequent follicular atresia. The combination of melatonin and vitamin D3 mitigates these deleterious effects by preserving follicular structure and restoring hormonal balance. This combination may represent an effective fertility-preserving strategy for individuals undergoing gonadotoxic treatments. Future studies should focus on determining the optimal administration protocols for melatonin and vitamin D3, performing a comprehensive analysis of the underlying molecular mechanisms, and exploring the impact of alternative forms of programmed cell death on ovarian function and reproductive outcomes. Additionally, further research is warranted to evaluate the clinical applicability of this combination therapy in oncofertility settings.

## Figures and Tables

**Table 1:** List of investigated genes and primers.

Gene Name	PCR Forward Primer	PCR Reverse Primer	Product Length
RAT-PTEN	AAAGCTGGGAAAGGACGGAC	GCGCCTCTGACTGGGAATAG	147
RAT-AMH	CCCGCTATTTGGTGCTGACT	GCGTGAAACAGCGGGAATC	153
RAT-FOXO3a	CTCTGGGCAACCGAGGAAAT	ATCTGGGACAAAGTGAGCCG	120
RAT- GAPDH	AAGAAGGTGGTGAAGCAGGC	TCCACCACCCTGTTGCTGTA	203

## Data Availability

The original contributions presented in this study are included in the article. Further inquiries can be directed to the corresponding author.
